# Food Insecurity and the Nexus of Co-Occurring Parental Distress and Child Internalizing and Externalizing Behavior Problems

**DOI:** 10.1007/s10802-026-01444-z

**Published:** 2026-03-16

**Authors:** Jun-Hong Chen, Jesse J. Helton, Michael G. Vaughn, Julie Birkenmaier, Shuya Yin, Cao Fang, Chien-Jen Chiang, Chi-Fang Wu, Yuanyuan Yang

**Affiliations:** 1 Saint Louis University, Saint Louis, MO USA; 2 Washington University in St. Louis, 1 Brookings Dr., Saint Louis, MO 63130 USA; 3 University of Texas at San Antonio, San Antonio, TX USA; 4 University of Illinois at Urbana- Champaign, Urbana, IL USA; 5 The University of Oklahoma, Norman, USA

**Keywords:** Food insecurity, Psychological distress, Problematic behaviors, Generalized propensity score weight

## Abstract

Parental psychological distress and children’s internalizing and externalizing behavioral problems could co-occur rather than operate independently, yet it remains unclear whether the likelihood of such co-occurrence varies by levels of food insecurity. By examining these co-occurring challenges in the context of food insecurity, this study aims to clarify the extent to which the alleviation of food insecurity could reduce the co-occurrence of parental psychological distress and child internalizing and externalizing behaviors. Using the 2019 and 2020 data waves of the Panel Study of Income Dynamics (*N* = 1,196), this study firstly conducts a latent profile analysis to investigate heterogeneity of co-occurrence patterns. Next, this study utilizes a multinomial logistic regression model to investigate the impacts of food insecurity on the risk of different co-occurrence patterns. To address selection bias, generalized propensity score weighting is applied to ensure demographic characteristics are similar to each other across different food insecurity levels (i.e., non-significant difference). Results show that, compared to families living with low/very low food insecurity, families living with high food security experience lower risk of co-occurrence of parental psychological distress and child internalizing and externalizing problematic behaviors (relative risk ratio = 0.41, *p* < 0.05). For practical implications, these findings suggest that lower risk of the co-occurrence of psychological distress in parents as well as internalizing and externalizing behavioral difficulties in children are more likely to be observed among families living with high food security.

## Introduction

In 2022, the U.S. Department of Agriculture (USDA) reported that 3.3 million American households with children experienced food insecurity, including difficulty affording food and lacking the financial resources necessary to maintain balanced meals (Rabbitt et al., 2023). This issue is deeply concerning not only for its prevalence but also for its substantial impact on children and their families. A growing body of research has identified food insecurity as a significant contributor to child internalizing and externalizing behavioral problems (Burke et al., 2016; Gallegos et al., 2021; Jackson & Vaughn, 2017; Kimbro & Denney, 2015). If left unaddressed, these behavioral difficulties may have long-term consequences for children’s well-being, including challenges in establishing stable relationships with others and diminished earnings when turning into adulthood (Vergunst et al., 2023). To support both prevention and intervention efforts, it is essential to clarify the association between food insecurity and children’s behavioral problems.

### Food Insecurity

When evaluating the impacts of socioeconomic adversities, studying food insecurity rather than relying solely on income or other socioeconomic indicators is critically important because food insecurity captures the lived and day-to-day consequences of economic hardship that income measures often miss (Banks et al., 2021). Different from other socioeconomic indicators such as income or housing instability that reflects long-term structural constraints, food insecurity reflects a more immediate and tangible form of hardship that directly affects daily survival (Norris et al., 2023). While low-income status or unemployment signals economic disadvantage, these conditions do not always result in instant or severe consequences, as households may rely on savings, social support, or temporary assistance to buffer short-term financial strain. In contrast, food insecurity indicates that these coping mechanisms have already been exhausted, meaning individuals and families are experiencing an actual lack of food or uncertainty about their next meal (McKernan, 2008 ). This makes food insecurity a more concrete and urgent manifestation of socioeconomic adversity, as it represents not just limited resources in theory, but real deprivation in practice, with immediate implications for physical health, mental well-being, and daily functioning (Hawkins & Panzera, 2021 )

### Food Insecurity and its Associations with Child Behavioral Outcomes

A growing body of empirical research underscores the substantial influence of food insecurity on child behavioral development. Shankar et al. (2017) demonstrate that inadequate access to food can meaningfully disrupt children’s emotional regulation, placing them at elevated risk for various emotional difficulties relative to their food-secure peers. Consistent with this pattern, Thomas et al. (2019) find that food insecurity significantly heightens children’s vulnerability to depressive disorders. Lu et al. (2019) further report a notable association between food insecurity and the emergence of hyperactivity-related symptoms. Complementing these behavioral findings, neurobiological evidence presented by Royer et al. (2021) indicates that food insecurity can impede key cognitive and socioemotional processes that are foundational for healthy behavioral development. Taken together, these empirical studies highlight a robust relationship between food insecurity and adverse child behavioral outcomes.

### Food Insecurity and its Associations with Parental Psychological Distress

Notably, parents living with food insecurity could experience elevated psychological distress as well (Cain, 2022). Such caregiver distress can negatively influence children’s developmental outcomes because these stressors may undermine parents’ capacity to provide consistent, nurturing, and cognitively stimulating care, while increasing the likelihood of harsh or inconsistent discipline and interparental conflict (Masarik & Conger, 2017). Disruptions in these family functioning processes may create an environment of instability and insecurity for children, hindering their socioemotional adjustment and contributing to the emergence of problematic behaviors. In turn, children’s problematic behaviors can heighten parental mental health risks; for example, Breaux and Harvey (2019) found that child hyperactivity symptoms predict greater depressive symptoms among caregivers. Taken together, this body of research suggests that, in the context of food insecurity, both parental psychological distress and child problematic behaviors could be elevated.

### Social Determinants of Health Framework

Food insecurity and its associations with parental and child outcomes can be elaborated in the social determinants of health framework. More specifically, based on social determinants of health framework, food insecurity functions not only as a material deprivation but as a chronic psychosocial stressor that alters children’s emotional and behavioral regulation systems (Norris et al., 2023). Uncertainty about food availability activates prolonged stress responses, contributing to dysregulation of the hypothalamic–pituitary–adrenal axis and heightened physiological reactivity. Such stress-related neurobiological alterations compromise executive functioning processes, including attention control, inhibitory regulation, and cognitive flexibility, that are foundational to adaptive behavior (Tarullo et al., 2020; Wesarg et al., 2020). In addition to children, food insecurity could also result in parental psychological distress, given that it represents a form of material hardship that systematically constrains parents’ ability to meet t basic nutritional needs (Cain et al., 2022). Such strain increases vulnerability to psychological issues, such as depressive symptoms, anxiety, and emotional exhaustion. Heightened psychological distress may, in turn, tax parents’ cognitive and emotional resources, worsening emotional availability (Masarik & Conger, 2017). Therefore, among families experiencing food insecurity, it is possible that both children and their parents could be affected.

### Co-Occurrence of Parental Psychological Distress and Child Behavioral Problems

Notably, evidence indicates that parental psychological distress and children’s internalizing and externalizing problems often co-occur rather than operate independently within socioeconomically vulnerable families (Zhu et al., 2023). This co-occurrence is consistent with family systems perspectives, which emphasize that socioeconomic adversities rarely affect parents or children in isolation but instead permeate the broader family ecology (Zhu et al., 2023). The co-occurrence of parental psychological distress and child behavioral problems can be understood as the result of dynamic and reciprocal processes within the family system (Wiggins et al., 2015). Parental psychological distress, such as depression, anxiety, or chronic stress, can impair caregivers’ emotional availability, consistency, and responsiveness, which in turn may increase children’s risk for emotional dysregulation and behavioral difficulties (Ribas et al., 2024). At the same time, children’s behavioral problems can elevate parenting demands and emotional burden, thereby exacerbating parental stress and psychological symptoms (O’leimat et al., 2019). Over time, repeated exposure to these interacting stress processes can lead to stable patterns in which parental distress and child behavioral problems reinforce one another, resulting in their frequent co-occurrence across development.

### Research Gaps

Empirical evidence has investigated the influences of food insecurity on child behavioral outcomes as well as parental psychological distress. Yet, among these studies, it remains unclear whether food insecurity specifically functions as a risk factor that precipitates the simultaneous occurrence of parental psychological distress and child internalizing and externalizing behavioral problems. Clarifying the relationship between food insecurity and the joint occurrence of parental distress and child problematic behaviors is crucial, as such insights can reveal whether risk of the co-occurrence of psychological distress in parents as well as internalizing and externalizing behavioral difficulties in children could be lower in families living with higher food security, a key for practical implications.

### Current Study

The aim of this study is to clarify the impacts of food insecurity on the risk of co-occurrence of parental psychological distress and child behavioral issues. Recognizing that people may exhibit varying patterns of co-occurrence, this study first investigates the heterogeneity in the co-occurrence patterns. Next, this study investigates the impacts of food insecurity on the risk of experiencing co-occurrence patterns applied with propensity score weight to support causal inference. By examining the co-occurrence of these child and parental challenges in the context of food insecurity, the findings are anticipated to offer further insights to clarify to what extent that food insecurity is associated with the co-occurrence of parental psychological distress and child internalizing and externalizing behaviors.

## Method

### Data

This study employs data from children and their families through the 2019 Panel Study of Income Dynamics (PSID) Main Study (Panel Study of Income Dynamics, 2019) as well as 2020 Child Development Supplements (CDS). The 2019 PSID Main Study collected extensive data on household economic well-being and daily needs, including measures of 2019 food insecurity, household income, and wealth. It also provided caregiver demographic information, such as age, gender, race, educational attainment, and employment status. The 2020 CDS provided additional data on 2020 caregiver psychological distress as well as children’s internalizing and externalizing behaviors. By combining data from the PSID Main Study and the CDS, this dataset therefore establishes the temporal order that enables analyses to investigates food insecurity in 2019 and its associations with co-occurrence of parental psychological distress and child problematic behaviors in 2020.

This study draws from families completing the PSID Main Study as well as child behavior assessment interviews in the CDS. For families with multiple children, one child was randomly selected for inclusion in the analytic sample, resulting in a final sample of 1,196 children. The children had an average age of 7.4 years (SD = 2.8; min:3; max:12), with 49.2% were female. All caregivers were biological parents, 76.6% of whom were female, with an average age of 35.7 years (SD = 7.2).

IRB and informed consent statement were not applicable because all data utilized in this study are de-identified secondary data. There were no interactions or interventions with humans or animals in the current study.

### Measures

Food insecurity is a categorical variable that indicates whether a household experiences challenges related to food access, as measured by 18 items identified by the U.S. Department of Agriculture (Rabbitt et al., 2023). In the PSID, food insecurity is measured using the Household Food Security Survey Module developed by the USDA (Bickel et al., 2000). This instrument captures the extent to which households can reliably access and consume adequate and well-balanced meals, with higher total scores indicating greater severity of food insecurity. Households with 0 affirmative responses are classified as high food secure. Households with 1–2 affirmative responses are classified as marginally food secure, meaning that they generally have sufficient food but may experience occasional concerns or minor difficulties. Those reporting 3–7 affirmative responses are categorized as low food secure, while households with 8–18 affirmative responses fall into the very low food secure category, reflecting disrupted eating patterns and reduced food intake due to limited resources. For analytic purposes, the low and very low food secure categories were combined into a single food insecure group to enhance statistical power. To assess the robustness of findings based on categorical food insecurity defined by the USDA, we conducted a sensitivity analysis using continuous food insecurity scores. Results using continuous food insecurity scores are aligned with results using categorical food insecurity defined by the USDA, and the corresponding estimates are reported in Appendix Table 1.

**Table 1:** Descriptive results (*N* = 1,196)

Demographic features	High food secure (*n* = 893)	Marginal food insecure (*n* = 166)	Low/very low food insecure (*n* = 137)
Parent characteristics			
Age ***	36.1 (SD = 6.8)	34.9 (SD = 8.7)	33.6 (SD = 7.5)
Female *	73.9%	84.7%	84.3%
Race ***			
White American	63.9%	39.2%	38.0%
Black American	27.7%	49.4%	51.8%
Non-White, non-Black groups	8.4%	11.4%	10.2%
Unemployed *	24.0%	30.1%	30.7%
College or above **	23.0%	9.6%	4.4%
Baseline psychological distress ***	3.6 (SD = 3.0)	5.5 (SD = 3.7)	6.4 (SD = 4.3)
Child characteristics			
Age	7.4 (SD = 2.9)	7.3 (SD = 2.9)	7.6 (SD = 2.8)
Female	48.3%	50.4%	53.1%
History of behavioral problem *	11.8%	17.5%	19.0%
Family characteristics			
Single-parent family ***	23.2%	50.0%	50.5%
Household income ***	4.0 (SD = 3.7)	1.6 (SD = 1.0)	1.4 (SD = 1.1)
Household wealth ***	4.1 (SD = 12.7)	0.4 (SD = 2.7)	0.1 (SD = 2.3)

*p < 0.05 **p < 0.01 ***p < 0.001

Caregiver psychological distress is a continuous variable derived from the Kessler Psychological Distress Scale, which evaluates the severity of psychological distress based on a total score from items assessing feelings of nervousness, hopelessness, restlessness, exhaustion, sadness, and worthlessness (Kessler et al., 2002). Each item is measured using a 5-point scale, ranging from 0 (none of the time) to 4 (all of the time). The scale demonstrated acceptable internal consistency, with Cronbach’s alpha value is 0.74. Higher scores reflect greater psychological distress.

Child internalizing and externalizing behaviors are continuous variables, assessed using the Strengths and Difficulties Questionnaire developed by Goodman et al. (1998), with higher scores indicating more severe behavioral challenges. Both internalizing and externalizing behaviors were treated as continuous variables, reflecting the number of behavioral problems reported by parents using ten items for each domain (e.g., social withdrawal for internalizing and aggression for externalizing). The scale demonstrated acceptable internal consistency, with Cronbach’s alpha value is 0.81 and 0.77 for internalizing behaviors and externalizing behaviors, respectively.

To account for potential confounding effects due to factors that are also associated with parental psychological distress and child problematic behaviors, this study included covariates encompassing parent, child, and household characteristics reflecting 2019 information. Parent characteristics included age (in years), gender (male or female), employment status (employed or unemployed), educational attainment (college degree or higher). In the PSID, people consist of White American, African American, Hispanic, Indian American, Alaska Native, Native Hawaiian, and Pacific Islander. To have sufficient statistic power for analyses, these people are categorized into non-Hispanic White Americans, non-Hispanic Black Americans, and Non-White and non-Black groups. Child characteristics comprised age (in years), gender (male or female), and problematic behavior history, which is a binary indicator of whether the child had ever consulted a doctor or health professional for behavioral problems before the baseline (2019). Household characteristics included parenting structure (dual-parenting or single-parenting), household income, and household wealth. Household income was measured as a continuous variable, expressed as the ratio of household income to the U.S. federal poverty line adjusted for family size. Household wealth, which represents financial resources other than income, is a continuous variable measured by the ratio of household wealth to the US federal poverty line adjusted for family size. Based on data set availability, household wealth includes savings and other assets (e.g., farm or business, bonds, stocks, vehicles) minus all debts.

### Statistical Analyses

Building on the methodological framework outlined by Asparouhov and Muthén (2014), the present study applied a three-step procedure to investigate the extent to which food insecurity shapes the co-occurrence of parental psychological distress and children’s behavioral difficulties. In the initial step, a latent profile analysis was conducted to identify discrete subgroups of individuals characterized by distinct patterns of parental distress alongside child internalizing and externalizing behaviors. Individuals with similar co-occurrence patterns were grouped into the same class. It is important to note that classification in latent profile analysis does not imply that individuals within a class are identical; rather, it indicates that individuals with similar co-occurrence patterns are grouped into the same class (Nguefack et al., 2020; Ram & Grimm, 2009). The detection of two or more classes indicates there is heterogeneity in the co-occurrence patterns of parental psychological distress and children’s behavioral difficulties.

The second step entailed identifying the optimal number of latent classes by drawing on a comprehensive set of goodness-of-fit indices, including the log-likelihood value (LogL), Akaike’s Information Criterion (AIC), Bayesian Information Criterion (BIC), and the sample-size adjusted BIC (SABIC). Models characterized by higher LogL values and lower AIC, BIC, and SABIC values were considered to provide superior fit (Nylund et al., 2007; Chen et al., 2017). Classification accuracy was further evaluated using entropy, with higher entropy values indicating clearer delineation among classes. To more rigorously assess whether a model with ***k*** classes offered a statistically meaningful improvement over a model with ***k-1*** classes, the Vuong-Lo-Mendell-Rubin likelihood ratio test (VLMR) and the adjusted Lo-Mendell-Rubin test (ALMR) were employed; a significant p-value (*p* < 0.05) was interpreted as evidence favoring the more complex model. Consistent with recommendations by Nylund et al. (2007), to ensure adequate power for detecting differences between classes, each class should comprise at least 5% of the sample. All latent profile analyses and class enumeration procedures based on these criteria were conducted using Mplus 8.0. Collectively, these analytic steps facilitated the evaluation of whether the data supported the presence of two or more distinct co-occurrence patterns, thereby indicating heterogeneity in the joint presentation of parental psychological distress and child behavioral problems.

In the third step, as a categorical variable, the class membership that showed parent-child co-occurrence patterns was utilized as an outcome. A multinomial logistic regression model was applied to investigate the impacts of food insecurity on the risk of co-occurrence of parental psychological distress and child behavioral issues, while controlling for sociodemographic characteristics. The multinomial logistic regression model was conducted using the Stata 18.0 MP version. For variables used in our analyses, we observed no variance inflation factor (VIF) values greater than 10 (range: 1.02 to 1.51). This suggests no multicollinearity issue, which is in accordance with Vittinghoff et al. (2012).

For causal inference that accounts for potential selection bias in different food insecurity levels (i.e., high food security, marginal food security, low/very low food security), this study conducted analyses applied with generalized propensity score weighting. As a quasi-experimental method, generalized propensity score weighting was employed to ensure observations share similar demographic characteristics regardless of food insecurity levels. In such a case, it enables us to better assess the associations between food insecurity and its associations with parent-child co-occurrence patterns.

Following Guo & Fraser, (2014), we estimated generalized propensity score weights using a two-step procedure. First, a multinomial logistic regression model was estimated to predict the probability of membership in each food insecurity level (***Pₙ***), where ***n*** corresponds to the number of food insecurity levels. Demographic characteristics were included as covariates in the model. Parent covariates (age, gender, race, employment status, educational attainment, and baseline psychological distress), child covariates (age, gender, and history of behavioral issues), and household covariates (household income, household wealth, and family structure) were included as independent variables in the model used to predict (***Pₙ***). Second, the predicted probabilities (***Pₙ***) were used to construct inverse probability weights, defined as ***1/Pₙ***, to create the generalized propensity score weights. We then conducted balance diagnostics to evaluate whether application of generalized propensity score weighting makes these demographic features not statistically different between different levels of food insecurity.

Examination of missing data using Little’s test for missing completely at random (MCAR) and covariate-dependent diagnostics suggested that the data were missing at random (MAR), supporting the application of generalized propensity score weighting under MAR assumptions (Choi et al., 2019). All analyses were performed using full information maximum likelihood, which yields unbiased parameter estimates under MAR conditions.

## Results

### Descriptive Results

Table 1 presents the descriptive statistics comparing food-secure and food-insecure households. Parents in food-insecure households are younger (*p* < 0.05), more likely to be female (*p* < 0.01), and less likely to identify as White American (*p* < 0.001). They also experience higher rates of unemployment (*p* < 0.05), lower educational attainment (*p* < 0.001), and elevated levels of psychological distress at baseline (*p* < 0.001). For children, a higher prevalence of behavioural problem history is observed in food-insecure households (*p* < 0.01). Additionally, food-insecure families are more likely to be single-parent households (*p* < 0.001) and report lower household income (*p* < 0.001) and wealth (*p* < 0.001).

### Class Memberships

Class memberships were examined to explore the heterogeneity in co-occurrence of parental psychological distress and child behavioural issues. In Table 2, the VLMR and ALMR tests show that the model containing four or five classes did not fit the data in a significantly better way than the model containing three or two classes. For this reason, models containing two and three classes were considered. The LogL, AIC, BIC, and SABIC showed that the model containing three classes better fit the data than the model containing two classes. Along with the LogL, AIC, BIC, and SABIC indexes, the VLMR and ALMR tests also show that the model containing three classes fits the data in a significantly better way than the model containing two classes. Therefore, this study adopts the three-class model to determine class memberships.

**Table 2:** Model indicators of latent profile analysis (*N* = 1,196)

Classes	LogL	Entropy	AIC	BIC	Adj. BIC	VLMR (*p*)	ALMR (*p*)	Smallest Class (%)
2	−8640.894	0.830	17301.788	17352.656	17320.892	< 0.001	< 0.001	17.9
**3**	**−8553.326**	**0.859**	**17134.652**	**17205.866**	**17161.397**	**< 0.001**	**< 0.001**	**8.6**
4	−8492.011	0.842	17020.022	17111.583	17054.408	0.109	0.105	2.5
5	−8451.815	0.844	16947.630	17059.539	16989.658	0.647	0.652	1.8

*AIC* Akaike information criterion, *BIC* Bayesian information criteria, *LogL* loglikelihood, *VLMR* Vuong-Lo-Mendell-Rubin test. *ALMR* adjusted Vuong-Lo-Mendell-Rubin test. Higher values of entropy and lower values of AIC and BIC indicate better model fit. Significant VLMR and ALMR p-values indicate that k number of classes has a better fit than k-1 number of classes

**Fig. 1:**
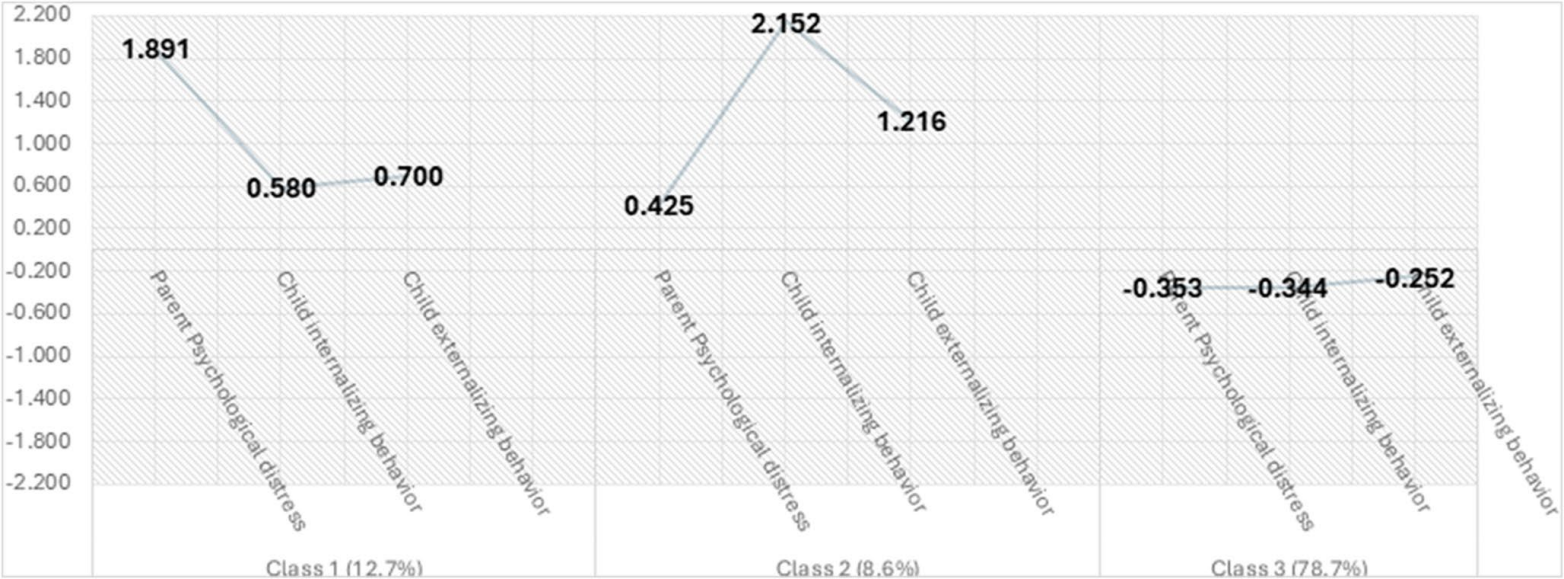
Standardized Scores of Parental Psychological Distress and Child Behavioral Issues by co-occurrence patterns (Class). Y-axis represents the standardized scores; [Class1]: Parent psychological distress is 1.89 standard deviations higher than the average; child internalizing and externalizing behaviors are 0.58 and 0.70 standard deviations higher than the average. [Class2]: Parent psychological distress is 0.42 standard deviations higher than the average; child internalizing and externalizing behaviors are 2.15 and 1.22 standard deviations higher than the average. [Class3]: Parent psychological distress is 0.35 standard deviations lower than the average; child internalizing and externalizing behaviors are 0.34 and 0.25 standard deviations lower than the average

Figure 1 illustrates the co-occurrence patterns of each class. Given different scales utilized by parental psychological distress and child problematic behaviors, the standardized scores were presented for comparable interpretation. Specifically, ***Class 1*** is characterized by high levels of both parental psychological distress and child internalizing and externalizing behaviors. Parental psychological distress in this group is 1.891 standard deviations higher than the overall average, while child internalizing and externalizing behaviors are 0.580 and 0.700 standard deviations higher than the average, respectively. Post-estimation results indicate that parental psychological distress in Class 1 is significantly more severe than the child behavioural issues, with a statistically significant difference (*p* < 0.05). ***Class 2*** also shows high levels of both parental psychological distress and child internalizing and externalizing behaviors. In this group, parental psychological distress is 0.425 standard deviations higher than the overall average, while child internalizing and externalizing behaviors are 2.152 and 1.216 standard deviations higher than the average, respectively. Post-estimation results show that child internalizing and externalizing behaviors in Class 3 are significantly more severe than parental psychological distress, with a statistically significant difference (*p* < 0.05). ***Class 3*** is characterized by low levels of parental psychological distress and low levels of child internalizing and externalizing behaviors. In this group, parental psychological distress is 0.353 standard deviations lower than the overall average, while child internalizing and externalizing behaviors are 0.344 and 0.252 standard deviations lower than the average, respectively.

In summary, ***Class 1*** is featured by “higher parental distress, high child behavioral problems.” ***Class 2*** is featured by “high parental distress, higher child behavioral problems.” ***Class 3*** is featured by low parental psychological distress and low child behavioral problems.

### Multinomial Logistic Regression Model Applied with Generalized Propensity Score Weighting

Results in Table 3 showed that higher food security is predictive of a lower probability of experiencing co-occurrence of parental psychological distress and child problematic behaviors. More specifically, relative to families in ***Class 3*** (characterized by low parental distress and low child problematic behaviors), families with high food security are significantly less likely to fall into ***Class 1*** (higher parental distress, high child behavioral problems) (relative risk ratio = 0.41, *p* < 0.05).

**Table 3:** Multinomial logistic model results *applied* ***with*** *generalized propensity score weight*

	Relative risk ratio of Class 1 to Class 3			Relative risk ratio of Class 2 to Class 3		
	Ratio	*p*-value	95% C.I.	Ratio	*p*-value	95% C.I.
Main predictor						
Low/very low food security (ref)	1.00			1.00		
Marginal food security	0.49	0.182	[0.17, 1.40]	0.92	0.887	[0.31, 2.76]
High food security	**0.41**	**0.032**	**[0.18**,** 0.93]**	0.39	0.890	[0.14, 1.12]
Parent covariates						
Age	0.97	0.202	[0.93, 1.02]	1.00	0.906	[0.94, 1.06]
Female	1.11	0.401	[0.16, 7.92]	1.93	0.082	[0.92, 4.06]
Race						
White American (ref)	1.00			1.00		
Black American	0.98	0.977	[0.43, 2.25]	0.73	0.447	[0.31, 1.74]
Non-White, non-Black groups	1.78	0.313	[0.58, 5.48]	2.93	0.056	[0.94, 9.12]
Unemployed	0.85	0.668	[0.40, 1.79]	**3.10**	**0.022**	**[1.23**,** 7.85]**
College or above	0.80	0.683	[0.28, 2.30]	**0.38**	**0.031**	**[0.16**,** 0.92]**
Baseline psychological distress	**1.42**	**< 0.001**	**[1.28**,** 1.58]**	**1.20**	**0.002**	**[1.07**,** 1.35]**
Child covariates						
Age	**1.16**	**0.017**	**[1.03**,** 1.30]**	1.06	0.371	[0.93, 1.22]
Female	0.96	0.896	[0.51, 1.79]	0.85	0.682	[0.39, 1.84]
History of behavioral problem	2.20	0.076	[0.92, 5.28]	**3.22**	**0.011**	**[1.31**,** 7.92]**
Family covariates						
Single-parent family	1.23	0.535	[0.59, 2.77]	**2.32**	**0.018**	**[1.15**,** 4.69]**
Household income	0.97	0.601	[0.85, 1.10]	1.03	0.718	[0.89, 1.18]
Household wealth	1.02	0.060	[0.99, 1.04]	1.01	0.476	[0.98, 1.04]

Class 1: “higher parental distress, high child behavioral problems.”

Class 2: “high parental distress, higher child behavioral problems.”

Class 3: Low Distress and Low Child Behavioral Problems

This study also observed that parental psychological distress at baseline is a risk factor predictive of a higher risk of experiencing ***Class 1*** co-occurrence pattern (higher parental distress, high child behavioral problems) (relative risk ratio = 1.42, *p* < 0.001) as well as a higher risk of experiencing ***Class 2*** co-occurrence pattern (high parental distress, higher child behavioral problems) (relative risk ratio = 1.20, *p* < 0.01). History of child behavioral issues is also a risk factor predictive of a higher risk of ***Class 2*** co-occurrence pattern (high parental distress, higher child behavioral problems) (relative risk ratio = 3.22, *p* < 0.05).

Table 4 shows that applying generalized propensity score weighting reduces demographic differences across food-insecurity levels to non-significance. By ensuring people in different food insecurity levels share similar demographic features, such a quasi-experimental approach is able to more validly assess the association between food insecurity and the co-occurrence of child behavioral problems and parental psychological distress.

**Table 4:** Balance Test of Propensity Score Weight (Demographic features comparison by food insecurity status) ^**a**^

	*P*-value before weighting	*P*-value after weighting ^b^
Parent characteristics		
Age	*P* = 0.025	*P* = 0.810
Female	*P* = 0.029	*P* = 0.426
Race/Ethnicity	*P* < 0.001	*P* = 0.530
Unemployed	*P* = 0.040	*P* = 0.592
College or above	*P* = 0.002	*P* = 0.060
Baseline psychological distress	*P* < 0.001	*P* = 0.437
Child characteristics		
Age	*P* = 0.337	*P* = 0.605
Female	*P* = 0.252	*P* = 0.348
History of behavioral problem	*P* = 0.024	*P* = 0.539
Family characteristics		
Single-parent family	*P* < 0.001	*P* = 0.600
Household income	*P* < 0.001	*P* = 0.508
Household wealth	*P* < 0.001	*P* = 0.392

^a^ By ensuring people share similar demographic features, such a balance test justifies the use of propensity score weight to get a valid causal inference of how food insecurity affects the risk of co-occurrence of caregiver psychological distress and child behavioral issues

^b^ After applying propensity score weight, this study found that households experiencing different levels of food insecurity share similar demographic features (i.e., no significant difference in all of these demographic features between households experiencing different levels of food insecurity)

## Discussion

Findings suggest that food insecurity is significantly associated with a higher risk of experiencing the co-occurrence pattern featured by high parental distress and high child problematic behaviors, even though household income is held as cone of covariates for constant. Such finding provides implications that addressing food insecurity could not only support basic material needs but also function as an upstream strategy that can strengthen family well-being by interrupting maladaptive parent-child dynamics. Findings of this study also imply that food insecurity plays a differentiated role depending on which component of the parent–child co-occurrence patterns is most pronounced. Specifically, in terms of the risk experiencing Class 1 situation (“higher parental distress, high child behavioral problems) rather than Class 3 situation (low parental psychological distress and low child behavioral problems), such risk is lower among people living with high food security compared to those living with low/very low food security. Notably, in terms of the risk experiencing Class 2 situation (“high parental distress, higher child behavioral problems”) rather than Class 3 situation (low parental psychological distress and low child behavioral problems), such risk does not significantly differ by food insecurity levels. These findings suggest that high food security is associated with lower risk of experiencing co-occurring family distress where parental psychological issues predominate, but not all kinds of co-occurring family distress. When child behavioral problems are more severe in co-occurring family distress, however, high food security is not significantly associated with such lower risk. Rather, it is possible that additional interventions, such as two-generation models that implies concurrent attention to parent and child, are required (Chase-Lansdale, & Brooks-Gunn, 2014). As this is not an intervention study, more efforts are thus required to directly examine the positive changes of high food security to child and family wellbeing.

In this study, we observed that Class 1 and Class 2 primarily differ in the relative prominence of parental psychological distress versus child behavioral problems, rather than reflecting fundamentally distinct co-occurrence patterns. To examine the robustness of our findings, we conducted a sensitivity analysis using a two-class model, although model fit indices favored the three-class solution. The two-class model identified two broad groups: [1] high parental psychological distress and child behavioral problems, and [2] low parental psychological distress and child behavioral problems. Results from this model indicated that higher food security was associated with a lower risk of belonging to the high-risk co-occurrence group. *However, relying solely on the two-class model may obscure important heterogeneity within high-risk families by treating all co-occurring distress as a single pattern*. In contrast, the three-class model allows for differentiation between family profiles in which parental psychological distress is more prominent versus those in which child behavioral problems are more severe. This distinction provides additional interpretive value, as our findings suggest that higher food security may be particularly protective in families where parental psychological distress predominates, but less effective when child behavioral problems are more severe. Such nuance offers important implications for practice, indicating that food security interventions alone may not be equally effective across all high-risk family profiles and that additional child-focused supports may be necessary for certain families. Accordingly, we retained the three-class model to better capture heterogeneity in co-occurring family distress and to inform more targeted intervention strategies.

Co-occurrence of parental psychological distress and child behavioral problems in the context of food insecurity may reflect a mutually reinforcing process of stress exposure within families. From this perspective, each latent class membership may reflect distinct ongoing reciprocal stress processes between parents and children, rather than discrete outcomes driven solely by food insecurity. For example, Class 2 membership might suggest that such reciprocal associations between parents and their children could result in worsening parental mental health and child behavioral problems, where parental mental health issues could be stronger than children’s behavioral issues. In the social determinate of health framework, food insecurity represents a chronic and unpredictable stressor that can directly heighten parental emotional strain through persistent concerns about meeting basic needs, financial instability, and feelings of inadequacy or guilt related to food provision (Engel, 2025). Elevated parental distress, in turn, may compromise emotional availability, consistency in parenting practices, and household routines, thereby increasing children’s vulnerability to behavioral and emotional difficulties (Masarik & Conger, 2017). Simultaneously, children’s behavioral problems may further intensify parental stress by increasing caregiving demands and emotional burden, particularly in resource-constrained environments (Wiggins et al., 2015). Through this reciprocal process, stress experienced by parents and children may accumulate and reinforce one another over time, contributing to the clustering of psychological distress and behavioral problems within families experiencing food insecurity.

Poverty is a multi-dimensional construct, encompassing not only limited access to adequate food but also other material and social deprivations, such as housing instability, unstable income, and limited access to healthcare or education (McKernan, 2008). These different dimensions of poverty can have distinct and overlapping effects on family functioning and psychological well-being (Jones et al., 2024). Therefore, focusing solely on food insecurity may underestimate the broader influence of poverty on child and family well-being. Future studies should consider the use of multiple dimensions of socioeconomic adversity using other methodologies such as measuring the cumulative financial adversities and identify its associations with child and parental health outcomes to inform more targeted interventions and public policies.

Findings of this study also suggest that parental psychological distress at baseline and a history of behavioral problems in the child are critical risk factors for the simultaneous occurrence of caregiver psychological distress and child behavioral issues. This co-occurrence highlights the need for early interventions that address both the emotional well-being of the caregiver and the behavioral health of the child. Moreover, these findings emphasize the importance of a comprehensive approach, as any of these two elements can lead to compounded difficulties, a risk factor impacting the family dynamic as a whole. Chase-Lansdale and Brooks-Gunn (2014) further support this perspective, highlighting the critical need for interventions that improve both child and caregiver outcomes, particularly in economically vulnerable families, where these two factors are interconnected.

While policies such as Supplemental Nutrition Assistance Program aim to combat food insecurity, they still face significant barriers and limitations. The social benefit application process itself is often seen as a bureaucratic burden, contributing to “a service gap”, namely a cycle in which households lose benefits temporarily as they wait to re-establish eligibility, thus exacerbating hardships faced by people in need and causing financial harm on governments given the increase in administrative costs (Homonoff & Somerville, 2021; Wagner & Huguelet, 2016). Recent research calls for greater flexibility in the application and eligibility to better serve vulnerable populations (Giannella et al., 2024; Ettinger et al., (2019). From a holistic perspective, the delivery of regular quality food in conjunction with assessment and treatment of parent and child mental health, and positive parenting intervention targeted toward food insecure households may ameliorate the intertwined risks observed (Althoff et al., 2016; Masarik & Conger, 2017).

It is instructive to situate the present findings alongside prior studies using the Panel Study of Income Dynamics to clarify this study’s distinct contribution to the literature on food insecurity and child well-being. Earlier analyses by Chen et al., (2024) examined trajectories of food insecurity from 2014 to 2018 and their associations with child internalizing and externalizing behaviors in 2019, demonstrating that cumulative exposure to hardship has lasting consequences and that improvements in food insecurity may not fully offset the effects of earlier deprivation. In related work done by Chen et al. (2026), it has been reported that there are racial disparities in the family stress process, where parental distress in 2020 as a mediator linking 2019 food insecurity and 2021 child hyperactivity, highlighting variation in the pathways through which hardship affects child development. Together, these studies emphasize longitudinal risk exposure and mediation processes.

In contrast, the present study shifts attention from temporal trajectories and pathway mediation to the contemporaneous family system. Using a person-centered latent profile approach, we identify distinct constellations of co-occurring parental psychological distress and child behavioral problems in 2020 and examine how these profiles vary by prior food insecurity status. This approach reveals that food insecurity is not uniformly associated with all forms of family distress; instead, it is most strongly linked to a profile characterized primarily by elevated parental distress. Such heterogeneity would likely remain obscured in traditional variable-centered or trajectory-based analyses. By highlighting how material hardship clusters with caregiver and child well-being within families, this study extends prior work and suggests that addressing food insecurity is critical not only for meeting basic needs but also for improving caregiver mental health and child adjustment simultaneously, more than just separately.

It is important to interpret the present findings within the broader context of the COVID-19 pandemic, a period marked by substantial social disruption, economic instability, school closures, and heightened psychological stress for families. These unprecedented conditions may have altered family routines, access to resources, and mental health processes in ways that differ from non-pandemic contexts. As a result, associations observed in this study may reflect not only underlying family risk processes but also pandemic-related stressors that intensified existing vulnerabilities. Considering the unique circumstances of COVID-19 is therefore essential when interpreting the magnitude and generalizability of the findings, as relationships identified during this period may not fully generalize to post-pandemic conditions. Acknowledging this context underscores the need for future research to examine whether similar patterns persist beyond periods of widespread societal disruption.

While this study adds to the understanding of food insecurity and its impacts on the risk of co-occurrence of child behavioral problems and parental psychological distress, limitations are still present. For example, because both parental psychological distress and child behavioral problems were parent-reported, shared method variance may have influenced observed associations, and parental distress may have shaped perceptions of child behavior. These concerns may be heightened during the COVID-19 pandemic. Although latent class analysis is informative, class structures identified under these conditions may have limited generalizability. Findings should therefore be interpreted with caution. Also. the findings are based on the PSID dataset, which might not fully represent the experiences of populations in different cultural settings across different countries. In addition, there are likely unmeasured confounding factors, such as genetic-based biological information. Stable schooling is also an important factor determinant of child and family well-being. As a benchmark laid by this study, future studies are encouraged to include more comprehensive measures of these characteristics to investigate whether the impacts of food insecurity on the risk of co-occurrence could vary by these characteristics.

## Conclusions

Our findings suggest that addressing food insecurity could be conducive to lowering the risk of co-occurrence of parental distress and child behavioral problems. These findings deepen the understandings of benefits of ensuring stable access to food security, not only for material needs but also for a healthy family well-being by interrupting maladaptive parent-child dynamics. Interventions that stabilize household basic material needs via food assistance, may therefore function as upstream determinants of child and parental well-being simultaneously.

## Data Availability

Not applicable.

## Appendix 1: Multinomial logistic model (using continuous food insecurity scores)

**Table:**

	Relative risk ratio of Class 1 to Class 3	Relative risk ratio of Class 2 to Class 3
Main predictor		
Food insecurity scores	**1.26****	1.03
Parent covariates		
Age	0.99	1.0
Female	1.80	1.28
Race		
White American (ref)	1.00	1.00
Black American	0.68	0.72
Non-White, non-Black groups	1.82	2.25
Unemployed	0.82	1.33
College or above	0.64	**0.67****
Baseline psychological distress	**1.35*****	**1.18*****
Child covariates		
Age	1.01	1.05
Female	1.15	0.94
History of behavioral problem	1.45	**3.94*****
Family covariates		
Single-parent family	0.86	1.41
Household income	0.97	0.98
Household wealth	1.05	1.00

Class 1: “higher parental distress, high child behavioral problems.”

Class 2: “high parental distress, higher child behavioral problems.”

Class 3: Low Distress and Low Child Behavioral Problems

**p*<0.05 ***p*<0.01 ****p<0.001*

## References

[CR1] Althoff, R. R., Ametti, M., & Bertmann, F. (2016). The role of food insecurity in developmental psychopathology. *Preventive Medicine,**92*, 106–109. 10.1016/j.ypmed.2016.08.012 27514244 10.1016/j.ypmed.2016.08.012 PMC5085882

[CR2] Asparouhov, T., & Muthén, B. (2014). Auxiliary variables in mixture modeling: Three-step approaches using Mplus. *Structural Equation Modeling: A Multidisciplinary Journal*, *21*(3), 329–341. 10.1080/10705511.2014.915181

[CR3] Banks, A. R., Bell, B. A., Ngendahimana, D., Embaye, M., Freedman, D. A., & Chisolm, D. J. (2021). Identification of factors related to food insecurity and the implications for social determinants of health screenings. *BMC Public Health,**21*(1), Article 1410. 10.1186/s12889-021-11465-6 34271906 10.1186/s12889-021-11465-6 PMC8284017

[CR46] Bickel, G., Nord, M., Price, C., Hamilton, W., & Cook, J. (2000). Guide to measuring household food security, Revised 2000.

[CR4] Breaux, R. P., & Harvey, E. A. (2019). A longitudinal study of the relation between family functioning and preschool ADHD symptoms. *Journal of Clinical Child and Adolescent Psychology,**48*(5), 749–764. 10.1080/15374416.2018.1437737 29578799 10.1080/15374416.2018.1437737 PMC12188987

[CR5] Burke, M. P., Martini, L. H., Çayır, E., Hartline-Grafton, H. L., & Meade, R. L. (2016). Severity of household food insecurity is positively associated with mental disorders among children and adolescents in the United States. *Journal of Nutrition,**146*(10), 2019–2026. 10.3945/jn.116.232298 27581581 10.3945/jn.116.232298

[CR6] Cain, K. S., Meyer, S. C., Cummer, E., et al. (2022). Association of food insecurity with mental health outcomes in parents and children. *Academic Pediatrics,**22*(7), 1105–1114. 10.1016/j.acap.2022.04.010 35577282 10.1016/j.acap.2022.04.010 PMC10153634

[CR7] Chase-Lansdale, P. L., & Brooks-Gunn, J. (2014). Two-generation programs in the twenty-first century. *Future of Children,**24*(1), 13–39. 10.1353/foc.2014.0003 25518701 10.1353/foc.2014.0003

[CR8] Chen, J-H., Huang, C-H., Wu, C-F., Jonson-Reid, M., & Drake, B. (2024). The application of family stress model to investigating adolescent problematic behaviors: The moderating role of assets. *Journal of Family and Economic Issues,**45*(1), 174–183. 10.1007/s10834-023-09902-2 10.1007/s10834-023-09902-2 PMC10139908 37360657

[CR9] Chen, Q., Luo, W., Palardy, G. J., Glaman, R., & McEnturff, A. (2017). The efficacy of common fit indices for enumerating classes in growth mixture models when nested data structure is ignored: A Monte Carlo study. *SAGE Open,**7*(1), Article 2158244017700459. 10.1177/2158244017700459

[CR10] Choi, J., Dekkers, O. M., & le Cessie, S. (2019). A comparison of different methods to handle missing data in the context of propensity score analysis. *European Journal of Epidemiology,**34*(1), 23–36. 10.1007/s10654-018-0447-z 30341708 10.1007/s10654-018-0447-z PMC6325992

[CR12] de Ettinger Cuba, S., Chilton, M., Bovell-Ammon, A., et al. (2019). Loss of SNAP is associated with food insecurity and poor health in working families with young children. *Health Affairs,**38*(5), 765–773. 10.1377/hlthaff.2018.05265 31059367 10.1377/hlthaff.2018.05265

[CR13] Engel, K. (2025). Parenting stress in households experiencing food insecurity: Mental health as a mediator? *Maternal and Child Health Journal,**29*(9), 1244–1252. 10.1007/s10995-025-04131-5 40690156 10.1007/s10995-025-04131-5 PMC12460468

[CR14] Gallegos, D., Eivers, A., Sondergeld, P., & Pattinson, C. (2021). Food insecurity and child development: A state-of-the-art review. *International Journal of Environmental Research and Public Health,**18*(17), Article 8990. 10.3390/ijerph18178990 34501578 10.3390/ijerph18178990 PMC8431639

[CR15] Giannella, E., Homonoff, T., Rino, G., & Somerville, J. (2024). Administrative burden and procedural denials: Experimental evidence from SNAP. *American Economic Journal: Economic Policy*, *16*(4), 316–340. 10.1257/pol.20220701

[CR16] Goodman, R., Meltzer, H., & Bailey, V. (1998). The strengths and difficulties questionnaire: A pilot study on the validity of the self-report version. *European Child & Adolescent Psychiatry,**7*(3), 125–130. 10.1007/s007870050057 9826298 10.1007/s007870050057

[CR17] Guo, S., & Fraser, M. W. (2014). *Propensity score analysis: Statistical methods and applications*. SAGE.

[CR18] Hawkins, M., & Panzera, A. (2021). Food insecurity: A key determinant of health. *Archives of Psychiatric Nursing,**35*(1), 113–117. 10.1016/j.apnu.2020.10.011 33593503 10.1016/j.apnu.2020.10.011

[CR19] Homonoff, T., & Somerville, J. (2021). Program recertification costs: Evidence from SNAP. *American Economic Journal: Economic Policy*, *13*(4), 271–298. 10.1257/pol.20190272

[CR20] Jackson, D. B., & Vaughn, M. G. (2017). Household food insecurity during childhood and adolescent misconduct. *Preventive Medicine,**96*, 113–117. 10.1016/j.ypmed.2016.12.042 28043828 10.1016/j.ypmed.2016.12.042

[CR21] Jones, D., Drake, B., Kim, H., Chen, J. H., Font, S., Putnam-Hornstein, E., & Jonson-Reid, M. (2024). Poverty indicators in the national child abuse and neglect data system child file: Challenges and opportunities. *Research on Social Work Practice*, *34*(3), 325–337. 10.1177/10497315231179658

[CR22] Kessler, R. C., Andrews, G., Colpe, L. J., et al. (2002). Short screening scales to monitor population prevalences and trends in non-specific psychological distress. *Psychological Medicine,**32*(6), 959–976. 10.1017/S0033291702006074 12214795 10.1017/s0033291702006074

[CR23] Kimbro, R. T., & Denney, J. T. (2015). Transitions into food insecurity associated with behavioral problems and worse overall health among children. *Health Affairs,**34*(11), 1949–1955. 10.1377/hlthaff.2015.0626 26526254 10.1377/hlthaff.2015.0626

[CR24] Lu, S., Perez, L., Leslein, A., & Hatsu, I. (2019). The relationship between food insecurity and symptoms of attention-deficit hyperactivity disorder in children: A summary of the literature. *Nutrients,**11*(3), 659. 10.3390/nu11030659 30893802 10.3390/nu11030659 PMC6470829

[CR25] Masarik, A. S., & Conger, R. D. (2017). Stress and child development: A review of the family stress model. *Current Opinion in Psychology,**13*, 85–90. 10.1016/j.copsyc.2016.05.008 28813301 10.1016/j.copsyc.2016.05.008

[CR26] McKernan, S. M. (2008). *Asset building and low income families*. The Urban Insitute.

[CR27] Nguena Nguefack, H. L., Pagé, M. G., Katz, J., Choinière, M., Vanasse, A., Dorais, M., Samb, O. M., & Lacasse, A. (2020). Trajectory modelling techniques useful to epidemiological research: A comparative narrative review of approaches. *Clinical Epidemiology,**12*, 1205–1222. 10.2147/CLEP.S265287 33154677 10.2147/CLEP.S265287 PMC7608582

[CR28] Norris, K., Jilcott Pitts, S., Reis, H., & Haynes-Maslow, L. (2023). A systematic literature review of nutrition interventions implemented to address food insecurity as a social determinant of health. *Nutrients,**15*(15), Article 3464. 10.3390/nu15153464 37571400 10.3390/nu15153464 PMC10421408

[CR29] Nylund, K. L., Asparouhov, T., & Muthén, B. O. (2007). Deciding on the number of classes in latent class analysis and growth mixture modeling: A Monte Carlo simulation study. *Structural Equation Modeling: A Multidisciplinary Journal,**14*(4), 535–569. 10.1080/10705510701575396

[CR30] O’leimat, A. S., Alhussami, M., & Rayan, A. (2019). The correlates of psychological distress among parents of children with psychiatric disorders. *Journal of Child and Adolescent Psychiatric Nursing,**32*(1), 24–32. 10.1111/jcap.12223 30693595 10.1111/jcap.12223

[CR31] Panel Study of Income Dynamics. (2019). *Panel Study of Income Dynamics public use dataset*. Institute for Social Research, University of Michigan. https://psidonline.isr.umich.edu

[CR32] Rabbitt, M. P., Hales, L. J., Burke, M. P., & Coleman-Jensen, A. (2023). *Household food security in the United States in 2022*. United States Department of Agriculture. Retrieved November 21, 2024, from http://www.ers.usda.gov/publications/pub-details/?pubid=107702

[CR33] Ram, N., & Grimm, K. J. (2009). Methods and measures: Growth mixture modeling: A method for identifying differences in longitudinal change among unobserved groups. *International Journal of Behavioral Development,**33*(6), 565–576. 10.1177/0165025409343765 23885133 10.1177/0165025409343765 PMC3718544

[CR34] Ribas, L. H., Montezano, B. B., Nieves, M., Kampmann, L. B., & Jansen, K. (2024). The role of parental stress on emotional and behavioral problems in offspring: A systematic review with meta-analysis. *Jornal de Pediatria,**100*(6), 565–585. 10.1016/j.jped.2024.02.003 38636551 10.1016/j.jped.2024.02.003 PMC11662746

[CR35] Royer, M. F., Guerithault, N., Braden, B. B., Laska, M. N., & Bruening, M. (2021). Food insecurity is associated with cognitive function: A systematic review of findings across the life course. *International Journal of Translational Medicine,**1*(3), 205–222. 10.3390/ijtm1030015

[CR36] Shankar, P., Chung, R., & Frank, D. A. (2017). Association of food insecurity with children’s behavioral, emotional, and academic outcomes: A systematic review. *Journal of Developmental and Behavioral Pediatrics,**38*(2), 135–150. 10.1097/DBP.0000000000000383 28134627 10.1097/DBP.0000000000000383

[CR38] Tarullo, A. R., Tuladhar, C. T., Kao, K., Drury, E. B., & Meyer, J. (2020). Cortisol and socioeconomic status in early childhood: A multidimensional assessment. *Development and Psychopathology,**32*(5), 1876–1887. 10.1017/S0954579420001315 33427182 10.1017/S0954579420001315 PMC7938639

[CR39] Thomas, M., Miller, D. P., & Morrissey, T. W. (2019). Food insecurity and child health. *Pediatrics,**144*(4), Article e20190397. 10.1542/peds.2019-0397 31501236 10.1542/peds.2019-0397

[CR40] Vergunst, F., Commisso, M., Geoffroy, M. C., Temcheff, C., Poirier, M., Park, J., Vitaro, F., Tremblay, R., Côté, S., & Orri, M. (2023). Association of childhood externalizing, internalizing, and comorbid symptoms with long-term economic and social outcomes. *JAMA Network Open,**6*(1), Article e2249568. 10.1001/jamanetworkopen.2022.49568 36622675 10.1001/jamanetworkopen.2022.49568 PMC9856729

[CR41] Vittinghoff, E., Glidden, D. V., Shiboski, S. C., & McCulloch, C. E. (2012). Predictor selection. In E. Vittinghoff, D. V. Glidden, S. C. Shiboski, & C. E. McCulloch (Eds.), *Regression methods in biostatistics: Linear, logistic, survival, and repeated measures models* (pp. 395–429). Springer US. 10.1007/978-1-4614-1353-0_10

[CR42] Wagner, J., & Huguelet, A. (2016). *Opportunities for states to coordinate Medicaid and SNAP renewals*. Washington, DC: Center on Budget and Policy Priorities. Retrieved November 21, 2024, from https://www.cbpp.org/sites/default/files/atoms/files/2-5-16fa.pdf

[CR43] Wesarg, C., Van Den Akker, A. L., Oei, N. Y. L., Hoeve, M., & Wiers, R. W. (2020). Identifying pathways from early adversity to psychopathology: A review on dysregulated hpa axis functioning and impaired self-regulation in early childhood. *European Journal of Developmental Psychology,**17*(6), 808–827. 10.1080/17405629.2020.1748594

[CR44] Wiggins, J. L., Mitchell, C., Hyde, L. W., & Monk, C. S. (2015). Identifying early pathways of risk and resilience: The codevelopment of internalizing and externalizing symptoms and the role of harsh parenting. *Development and Psychopathology,**27*(4 Pt 1), 1295–1312. 10.1017/S0954579414001412 26439075 10.1017/S0954579414001412 PMC4961476

[CR45] Zhu, X., Griffiths, H., & Murray, A. L. (2023). Co-developmental trajectories of parental psychological distress and child internalizing and externalizing problems in childhood and adolescence: Associations with self-harm and suicide attempts. *Research on Child and Adolescent Psychopathology*, *51*(6), 847–858. 10.1007/s10802-023-01034-3 36749476 10.1007/s10802-023-01034-3 PMC10195721

